# Spatiotemporal Dynamics of Sporadic Shiga Toxin–Producing *Escherichia coli* Enteritis, Ireland, 2013–2017

**DOI:** 10.3201/eid2709.204021

**Published:** 2021-09

**Authors:** Eimear Cleary, Martin Boudou, Patricia Garvey, Coilin Oh Aiseadha, Paul McKeown, Jean O’Dwyer, Paul Hynds

**Affiliations:** Technological University Dublin, Dublin, Ireland (E. Cleary, M. Boudou, P. Hynds);; Health Protection Surveillance Centre, Dublin (P. Garvey, P. McKeown);; Steeven’s Hospital, Dublin (C. Oh’Aiseadha);; University College Cork, Cork, Ireland (J. O’Dwyer)

**Keywords:** STEC, VTEC, spatial epidemiology, space-time scanning, sporadic infection, *Escherichia coli*, Shiga toxin, Shiga toxin–producing *E. coli*, verocytotoxin-producing *Escherichia coli*, clustering, bacteria, enteric infections, Ireland, food safety

## Abstract

The Republic of Ireland regularly reports the highest annual crude incidence rates of Shiga toxin–producing *Escherichia coli* (STEC) enteritis in the European Union, ≈10 times the average. We investigated spatiotemporal patterns of STEC enteritis in Ireland using multiple statistical tools. Overall, we georeferenced 2,755 cases of infection during January 2013–December 2017; we found >1 case notified in 2,340 (12.6%) of 18,641 Census Small Areas. We encountered the highest case numbers in children 0–5 years of age (n = 1,101, 39.6%) and associated with serogroups O26 (n = 800, 29%) and O157 (n = 638, 23.2%). Overall, we identified 17 space-time clusters, ranging from 2 (2014) to 5 (2017) clusters of sporadic infection per year; we detected recurrent clustering in 3 distinct geographic regions in the west and mid-west, all of which are primarily rural. Our findings can be used to enable targeted epidemiologic intervention and surveillance.

Over the previous decade, the Republic of Ireland has frequently reported the highest incidence rates of symptomatic Shiga toxin–producing *Escherichia coli* (STEC) infection in the European Union (EU) (*1*). The reported national crude incidence rate (CIR) of confirmed STEC infections in Ireland during 2017 was 923 cases (16.6 cases/100,000 population), equating to ≈10 times the EU average (1.66 cases/100,000 population) (*1*,*2*).

Shiga toxin–producing *E. coli* bacteria, of which there are >100 serotypes, were first discovered in 1977; the most well-known STEC strain, *E. coli* O157:H7, was first recognized as a pathogen in 1982. The Shiga toxin–producing group of *E. coli* includes serotypes O157, O26, and other enterohemorrhagic *E. coli* bacteria; serotypes are typically categorized by the presence of *stx1* or *stx2* genes (*3*). STEC is associated with a wide range of sequelae, from mild diarrhea to hemorrhagic colitis, hematochezia (bloody diarrhea), thrombotic thrombocytopenic purpura, and hemolytic uremic syndrome (HUS) causing intravascular lysis of red blood cells (*2*,*4*). Infection is characterized by several transmission routes, including consumption of contaminated food and water, person-to-person contact, or direct contact with infected animals (*4*,*5*). A recent study found the incidence of confirmed sporadic (i.e., nonoutbreak) STEC O157 infection in Ireland in 2008–2013 significantly elevated in regions characterized by high reliance on private groundwater (odds ratio [OR] 18.727; p<0.001) and high livestock densities (OR 1.001; p = 0.007) (*6*).

Transmission sources, pathways, and source–pathway interactions associated with STEC infection in Ireland are multifaceted, resulting in a complex exposure profile (*7*,*8*). Sporadic cases of infection are inherently difficult to attribute to specific risk factors for reasons that include the absence of accurate date-of-onset data, underreporting, misdiagnosis, myriad potential exposures, and surveillance limitations (*5*,*6*,*7*). Of 2,210 confirmed STEC cases reported in Ireland during 2008–2013, a total of 1,264 (57.2%) were defined as sporadic (*6*).

The high proportion of sporadic STEC infections relative to total annual cases in Ireland, and their association with environmental exposures, has made the spatiotemporal occurrence of STEC particularly important in public health. We used a suite of geostatistical approaches to explore spatiotemporal analyses of sporadic STEC infection in Ireland, a country characterized by the highest infection CIRs in Europe.

## Methods

### Data Collection and Processing

Because the primary study objective was to investigate patterns of domestic transmission, we excluded from analyses cases attributed to secondary infection (i.e., person-to-person transmission), and cases originating outside Ireland. We defined primary sporadic infection as a laboratory-confirmed case notified to a Department of Public Health during January 1, 2013–December 31, 2017, that had no reported epidemiological link to another notified case, or as an outbreak index case (i.e., the first documented case within a recognized cluster or outbreak). We obtained irreversibly anonymized case data from the Computerised Infectious Disease Reporting (CIDR) database (https://www.hpsc.ie/CIDR), a national database of notifiable infectious disease events reported by regional departments of public health in accordance with the Infectious Diseases (Amendment) Regulations 2011 (S.I. No. 452 of 2011).

Case confirmations were determined by both clinical and laboratory criteria; clinical criteria are primarily based on symptoms (diarrhea, abdominal pain, and HUS), and laboratory criteria require >1 of the following: isolation of strains positive for *stx1*, *stx2*, or both; direct detection of *stx1* or *stx2* nucleic acids (in the absence of strain isolation); or direct detection of Shiga toxin in fecal sample. We geographically referenced all confirmed cases to 1 of 18,641 Census Small Areas from the 2011 Central Statistics Office (CSO) census using the Health Atlas Ireland georeferencing tool. Small areas (SAs) are currently the smallest spatially defined area for census reporting in the state and exist as subdivisions within electoral districts (ED) of Ireland; each covers an area of 0.001–163 km^2^ and holds 80–120 dwellings. SAs are thus developed on the basis of household numbers and residential population (i.e., not spatial extent or population density) to report population-based statistics while ensuring personal and household anonymity. We delineated 3 infection subsets for additional analyses based upon epidemiologic and clinical significance: urban/rural classification; STEC serogroup (O157, O26, other); and case-patient age (<5 years, 6–65 years, >65 years)

**Figure 1:**
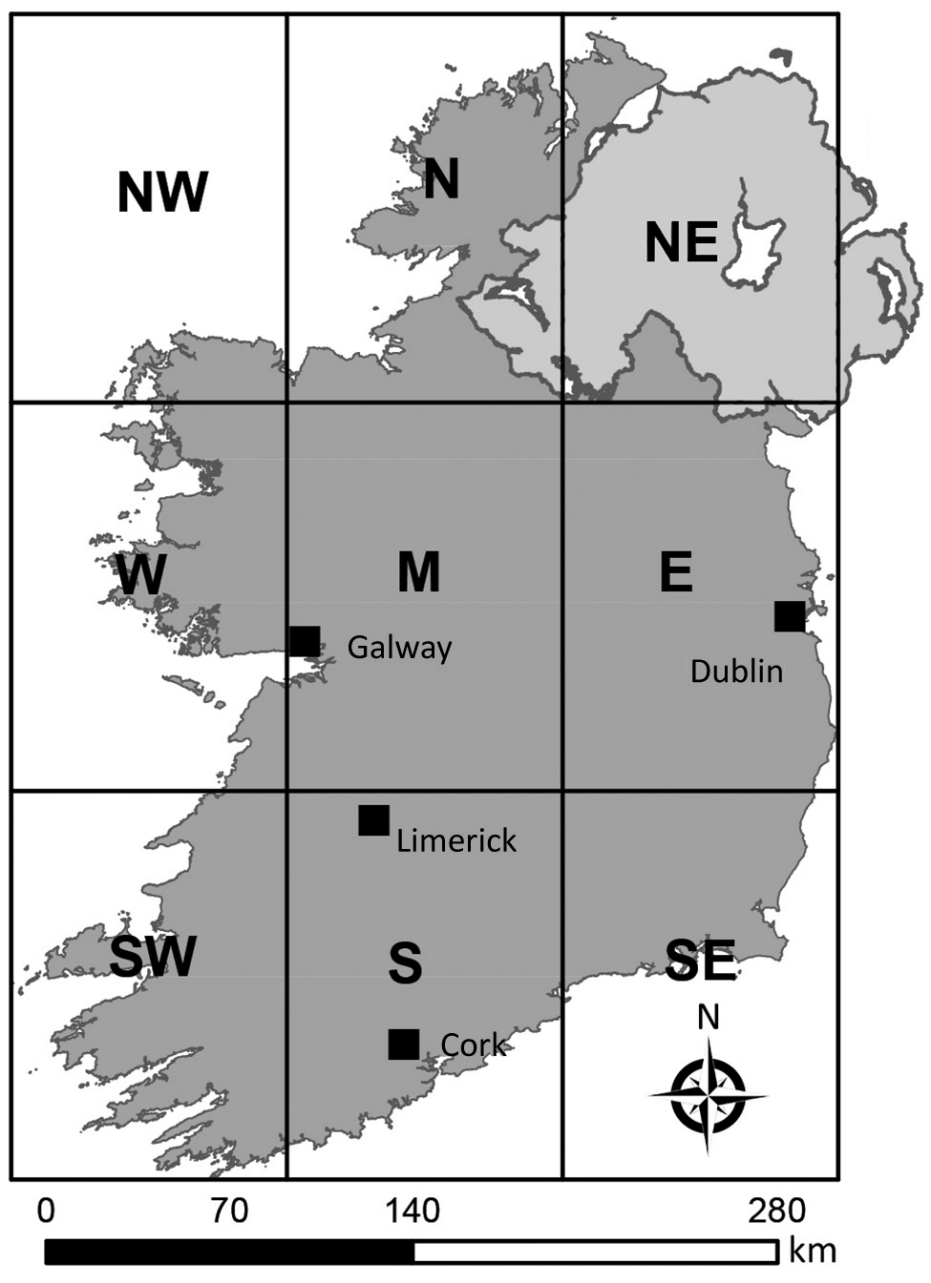
Geographic zones of Ireland. Sections of the grid represent the 8 distinct zones; zone NE, Northern Ireland, was not included in study of primary Shiga toxin–producing *Escherichia coli* enteritis cases. NW, northwest; N, north; W, west; M, midlands; E, east; SW, southwest; S, south; SE, southeast.

We extracted SA-specific human population counts from the 2011 and 2016 (CSO) census datasets and used those counts to calculate SA-specific STEC incidence rates. We merged the CSO’s 14 urban/rural categories to classify each spatial unit as rural or urban, using population density and settlement size used to verify classification. For reporting purposes, we have defined 8 distinct geographic zones in Ireland (Figure 1). Zone NE (Northern Ireland) is located outside CIDR surveillance boundaries and was not included for analyses. The Royal College of Physicians of Ireland Research Ethics Committee granted ethics approval for acquisition and analyses of human infection data (RCPI RECSAF_84).

### Seasonal Decomposition

We analyzed seasonal decomposition for monthly incidence rates using the seasonal-trend decomposition by LOESS (locally estimated scatterplot smoothing) (STL) method, which combines multiple regression with k–nearest neighbor meta-modeling (*9*). The STL method decomposes a time series into trend, seasonal, and residual components; we used an additive model for our study because peak values of the seasonal time-series exhibit a relatively constant trend (*10*). The monthly incidence rate (*Yv*) is equal to the sum of the trend (*Tv*) , the seasonal variation (*Sv*), and the residuals (*Rv*). For the seasonal decompositions, we used the STL() function in R version 3.6.0 (R Foundation for Statistical Computing, https://www.r-project.org).

### Spatial Autocorrelation (Anselin Local Moran’s *I*)

We used Anselin Local Moran’s *I* to examine individual features, specifically disease incidence within individual SAs, and their relationship to nearby features, returning localized clusters that may be correlated based on variance assigned to all individual spatial units (*11*). We calculated Local Moran’s I statistics using the cluster/outlier tool in ArcGIS software version 10.6 (ESRI, https://www.esri.com) and maps of resultant high and low spatial clusters generated. We used cluster/outlier analysis to classify statistically significant clusters of high values surrounded by high values; low values surrounded by low values; outliers of high values surrounded by low values; and low values surrounded by high values. We conceptualized spatial relationships using the inverse distance and Euclidean distance methods; we set significance at 95% based on pseudo p-values.

### Space-Time Scanning

We used SaTScan version 9.6 space-time cluster detection software (https://www.satscan.org) to identify temporally-specific high- and low-risk regions. We defined the space-time scan statistic by a cylindrical window with a circular (or elliptic) geographic base (e.g., radius unit) of which height corresponded to the time-period of potential clusters (*12*). The null test hypothesis presumes that geographic regions inside and outside the scanning area are characterized by an equal relative risk (RR) of infection during the analyzed time period. We compared RR differences using the log likelihood ratio (i.e., RR within an area is expected to be proportional to population size or population-years) (*13*). We used a discrete Poisson model due to the high level of geographic resolution (SA, n = 18,641) in our study (i.e., high number of SAs with 0 or 1 case over the modeled period). We used the total population of each SA from 2011 national census as a control parameter; we performed multiple scans to optimize parameter selection and outcome stability. We chose a maximum geographic cluster size of 10% of the population at risk to account for the low number of reported cases in many areas, in concurrence with a maximum cluster radius of 50 km. We aggregated data monthly; maximum temporal cluster duration was 3 months based on known seasonal effects. Cluster size was >10 reported cases to ensure that identified clusters contained enough observed cases.

We used findings from annual space-time scanning to acquire a spatiotemporal picture of recurrent cluster locations by a cluster recurrence index. We mapped each significant space-time cluster (p<0.05) from annual scans in ArcGIS software and attributed a binary cluster location (i.e., cluster membership (0/1) to each spatial unit (SA); the resulting cluster recurrence index value ranged from 0 (no clusters over study period) to 5 (>1 cluster per year over the study period).

Several tools have been developed to detect space-time anomalies, including the spatial varying temporal trend scan, implemented in SaTScan, which is used to identify unusual spatial cluster locations that contribute to substantial increase or decrease in general trends (*14*). The cluster recurrence index we describe aims to shed light on spatially specific, recurrent space-time hot spots of infection by providing an ordinal classification for all spatial units that may be amended over time and used for prospective surveillance purposes.

## Results

### Occurrence of Sporadic STEC Infection

**Figure 2:**
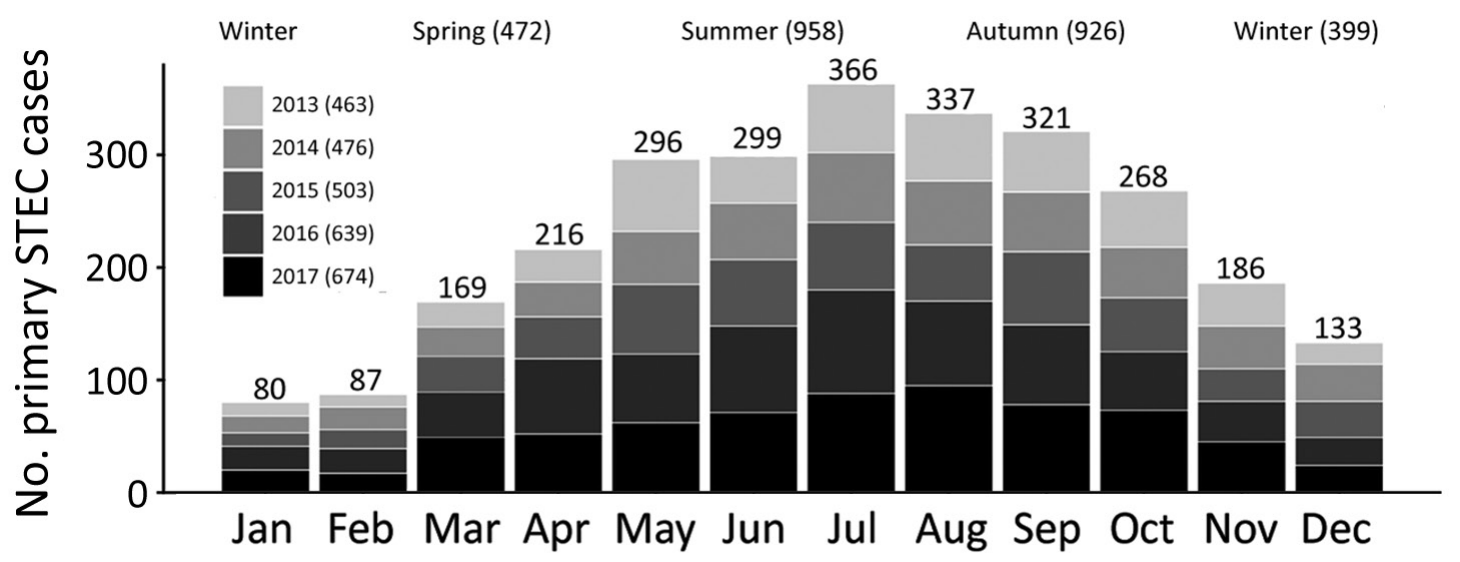
Temporal distribution of primary Shiga toxin–producing *Escherichia coli* (STEC) enteritis cases in Ireland, 2013–2017.

Of 2,783 confirmed sporadic cases included in the CIDR system during 2013–2017, we successfully geolinked 2,755 (98.9%) to a distinct spatial area. The most frequently confirmed serogroups associated with notified human infection were STEC O26 (n = 800, 29%) and O157 (n = 638, 23.2%) (Table 1; Appendix Table 1). We classified an additional 23.5% of confirmed infections as ungroupable (n = 391) or not serogrouped (n = 255). Of the remaining confirmed infection serogroups, STEC O145 (n = 126), O103 (n = 79), and O146 (n = 59) were the only serogroups associated with >50 confirmed infections. Temporal cumulative incidence rates exhibited an annual peak during late summer and early autumn; maximum peaks typically occurred during July (n = 366) (Figure 2). We observed yearly increase in case numbers between 2013 (463 cases) and 2017 (674 cases).

**Table 1:** Confirmed sporadic verocytotoxin-producing *Escherichia coli* infections in Ireland, 2013–2017*

Characteristic	VTEC O157	VTEC 026	Other serogroups	Not serotyped/ungroupable
Total	668	714	724	649
Age				
<5 y	231 (34.6)	431 (60.4)	255 (35.2)	184 (28.4)
6–64 y	373 (55.8)	232 (32.5)	314 (43.4)	273 (42.1)
>65 y	64 (9.6)	51 (7.1)	155 (21.4)	192 (29.6)
Setting				
Urban	288 (43.1)	278 (38.9)	329 (45.4)	309 (47.6)
Rural	380 (56.9)	436 (61.1)	395 (54.6)	340 (52.4)

*Values are no. (%) except as indicated. Percentages refer to VTEC serotype column totals vs case age range and Central Statistics Office Urban/Rural classification. VTEC, verocytotoxin-producing *Escherichia coli.*

**Figure 3:**
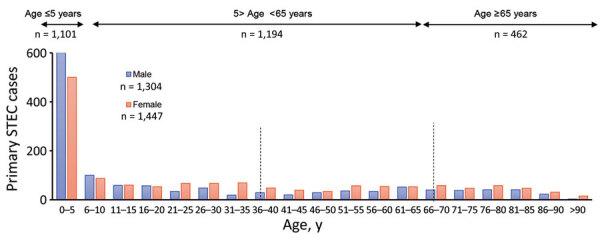
Distributions of primary STEC enteritis cases by age and sex, Ireland, 2013–2017. Dotted vertical lines show the main age-group divisions. STEC, Shiga toxin–producing *Escherichia coli*.

We observed markedly higher case numbers among children <5 years of age (Figure 3); 1,101 confirmed cases (39.6%) occurred within this subpopulation. Older persons (>65 years) were also disproportionately affected, accounting for 462 cases (16.6%, compared with 11.7% for the national population). A slightly higher rate of occurrence was associated with female patients (52.5%) than male (47.2%). We observed >1 cases more frequently in SAs classified as rural (1,252/6,242 SAs, 20.1%) than urban (n = 1,086/12,246 SAs, 8.9%) (Table 1). Pearson χ^2^ tests with Yates’ continuity corrections indicate a significant association between cases of STEC O26 infection and the <5 year age category (χ^2^ = 17.055; p<0.0001); STEC O26 cases were more than twice as likely to occur among this subpopulation than among those >5 years (OR 2.338). No statistical association was found between STEC serogroup and urban/rural classification (p = 0.6005) or the incidence of age-specific cases and urban/rural classification (p = 0.7803).

### Seasonal Decomposition

**Figure 4:**
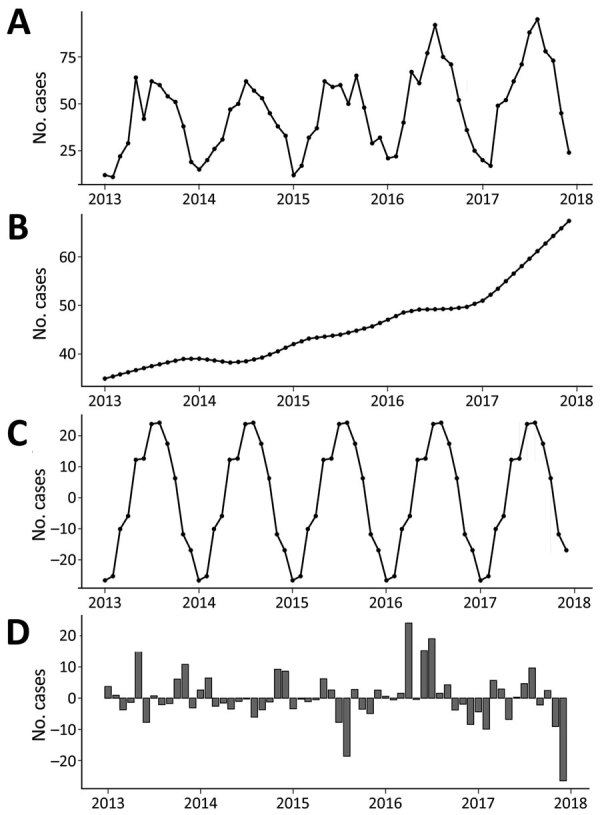
Trends and variations in confirmed primary Shiga toxin–producing *Escherichia coli* enteritis cases, Ireland, 2013–2017. A) all confirmed cases; B) decomposed 5-year trend of confirmed cases; C) seasonal variation in confirmed cases; D) calculated residual trend in confirmed cases.

The decomposed 5-year trend indicates a monotonic increase in the occurrence of sporadic infection, with a clear annual peak occurring during late summer (July–August) (Figure 4). Calculated residuals point to a relatively consistent annual and longer-term trend, ranging from a maximum of +24 cases during April 2016 to −26 cases during December 2018. Decomposed trends associated with <5 year and 6–64 year subcategories both exhibited an overall (nonmonotonic) increase; higher levels of variability were associated with the >65 year subcategory (Appendix Figure 1). We found substantial variation in seasonal infection peaks (all STEC serogroups) among delineated age categories; infections among the <5 year subpopulation peaked from May to July, whereas infections among the older subpopulation occurred in July–August, followed by a smaller secondary peak in October.

**Figure 5:**
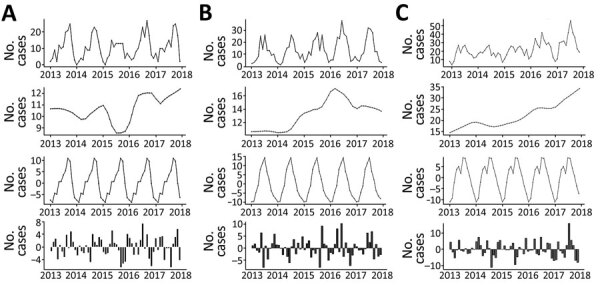
Trends and variations in confirmed cases of primary Shiga toxin–producing *Escherichia coli* enteritis in Ireland, 2013–2017, delineated for serogroup O157 (A), serogroup O26 (B), and other serogroups (C). Shown, top to bottom, are the trend for all confirmed cases, decomposed 5-year trend, seasonal variation, and calculated residual trend.

The general decomposed trend for STEC O157 infection indicates a relatively modest overall increase over the study period, with a marked decrease during 2015 (Figure 5). Conversely, the incidence of STEC O26 exhibited a greater increase from January 2013–April 2016, followed by a consistent decrease to the end of the study period. Other (non-O157 and non-O26) STEC serogroups exhibited a gradual monotonic increase over the study period. Seasonal signals indicate a notable difference between the 2 main serogroups; STEC O157 infections exhibit highest rates of occurrence during September–October, whereas STEC O26 notifications peak in July. Urban cases exhibited an annual peak from July–September, whereas categorically rural case notifications display a longer but decreasing peak from May–October (Appendix Figure 2).

### Spatial Autocorrelation (Local Anselin Moran’s *I*)

**Figure 6:**
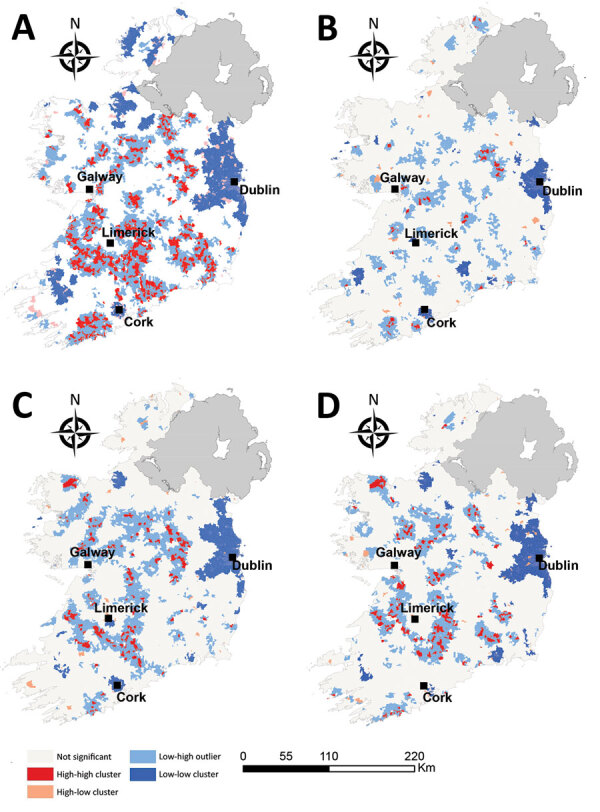
Spatial autocorrelation clusters of Shiga toxin–producing *Escherichia coli* (STEC) enteritis, Ireland, 2013–2017. A) All confirmed STEC infections; B) STEC O157 infections; C) STEC O26 infections; D) STEC infections in children <5 years.

The spatial distribution of high-high STEC incidence clusters were predominantly situated in zones S (south) and SE (southeast) around counties Clare, Limerick, and Tipperary (Figure 6), interspersed with smaller low-high outlier clusters. We observed infection cold spots (low-low clusters) around the greater Dublin area (zone E) and Cork city (zone S), in addition to counties Sligo (zone N) and Kerry (zone SW). The occurrence of STEC O157 infection clusters were geographically sparse with small distinct HH clusters (hotspots) observed in zones M, E, and S. We again observed large infection cold spots among the STEC O26 serogroup and the <5-year age group for all STEC in the urban centers of Dublin and Cork cities (zones E and S) in addition to counties Kerry, Waterford, and Sligo (zones SW, SE, and N). The spatial distribution of STEC O26 and <5-year age group hot spots of infection followed a similar trend to overall STEC clustering patterns: H-H clusters were identified in zones S, M, and E, in addition to 1 unique H-H cluster in zone NW, that we did not observe for STEC O157.

### Space-Time Scanning

**Figure 7:**
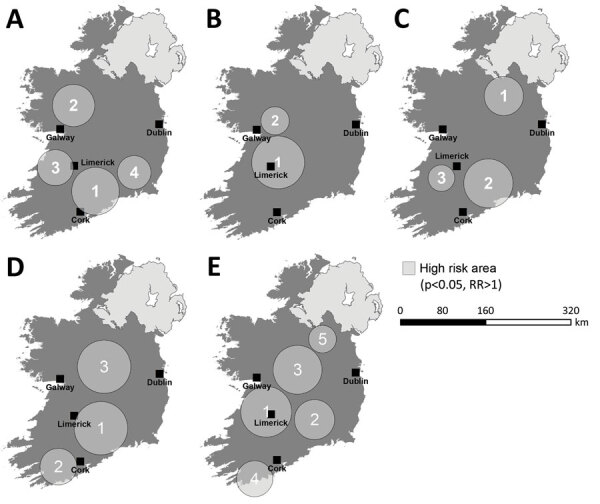
Annual space-time scanning of all confirmed primary Shiga toxin–producing *Escherichia coli* (STEC) enteritis cases in Ireland. A) 2013; B) 2014; C) 2015; D) 2016; E) 2017. Circles indicate clusters and numbers indicate the order in which they were identified during the study period. Clusters shown on the map have >10 confirmed cases, relative risk >1, p<0.05.

**Figure 8:**
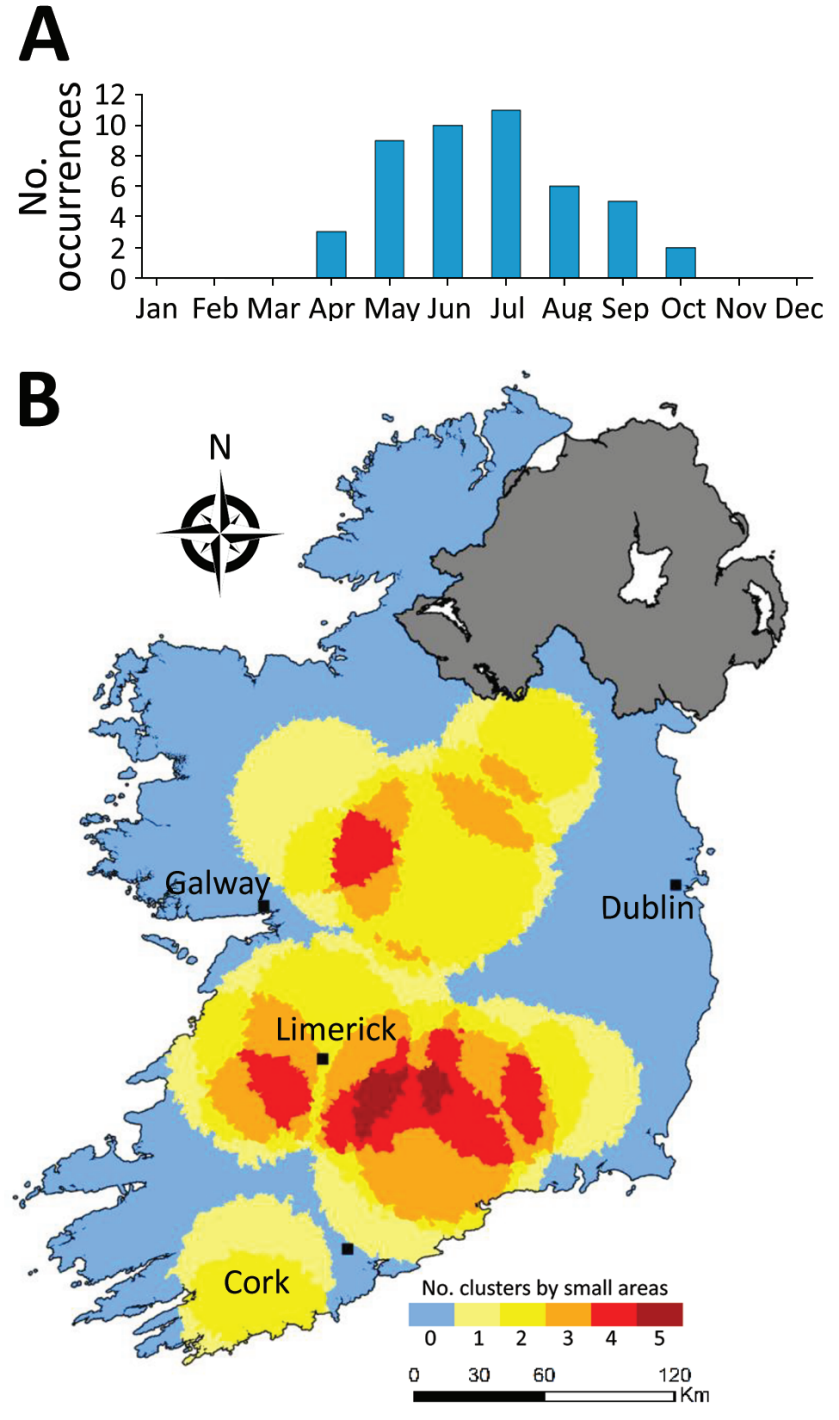
Monthly distribution of space-time clusters (A) and cluster recurrence index (0–5) within census small areas (B) for all confirmed primary Shiga toxin–producing *Escherichia coli* (STEC) enteritis cases in Ireland, 2013–2017.

Overall, we identified 17 distinct space-time clusters, ranging from 2 clusters during 2014 to 5 clusters during 2017 (Table 2; Figure 7). To acquire a clearer picture of hot- and cold spots relative to space-time cluster occurrence, we developed a space-time cluster recurrence index of 0 (SA never located within a space-time cluster) to 5 (SA situated within >1 space-time cluster during all study years) and generated maps of clusters (Figure 8). We identified 2 distinct areas situated southeast and southwest of Limerick City (Figure 1, zones M and S), and 1 area northeast of Galway city (zone M) as STEC infection hotspots during the study period. Of note, no major population centers other than Limerick were located within an identified hot spot; the entire eastern seaboard classified as an infection cold spot on the basis of population-adjusted incidence rates. Space-time clusters occurred from April–September and peaked during July (n = 11) (Figure 8).

**Table 2:** Results of year on year space-time scanning among all confirmed sporadic VTEC cases in Ireland, 2013-2017

Cluster no.	Population	No. cases	Expected	Observed/ expected	RR	Start date	End date	p value
2013								
1	268,082	32	4.62	6.93	7.37	2013 Jul 1	2013 Aug 31	0.00000000016
2	140,784	27	3.60	7.50	7.90	2013 May 1	2013 Jul 31	0.0000000041
3	147,000	25	3.76	6.65	6.97	2013 May 1	2013 Jul 31	0.00000030
4	154,425	18	3.91	4.61	4.75	2013 Apr 1	2013 Jun 30	0.022
2014								
1	370,743	40	9.72	4.11	4.40	2014 Jul 1	2014 Sep 30	0.00000022
2	75,467	14	1.96	7.15	7.34	2014 Apr 1	2014 Jun 30	0.0041
2015								
1	165,552	14	1.50	9.36	9.60	2015 Sep 1	2015 Sep 30	0.00018
2	245,189	27	6.79	3.97	4.14	2015 May 1	2015 Jul 31	0.00067
3	59,890	13	1.66	7.83	8.02	2015 May 1	2015 Jul 31	0.0037
2016								
1	299,166	42	10.59	3.97	4.18	2016 May 31	2016 Aug 30	0.00000033
2	119,941	21	4.20	5.00	5.14	2016 May 1	2016 Jul 30	0.0017
3	261,375	31	9.25	3.35	3.47	2016 Mar 31	2016 Jun 30	0.0044
2017								
1	345,279	54	12.93	4.18	4.45	2017 Jun 30	2017 Sep 29	0.00000000006
2	190,947	27	7.15	3.78	3.89	2017 Jul 30	2017 Oct 29	0.0042
3	232,749	29	8.71	3.33	3.43	2017 Jun 30	2017 Sep 29	0.015
4	66,817	15	2.47	6.06	6.18	2017 Apr 30	2017 Jul 29	0.017
5	81,564	10	1.00	10.04	10.18	2017 Sep 30	2017 Oct 29	0.027

*RR, relative risk; VTEC, verocytotoxin-producing *Escherichia coli*.

**Figure 9:**
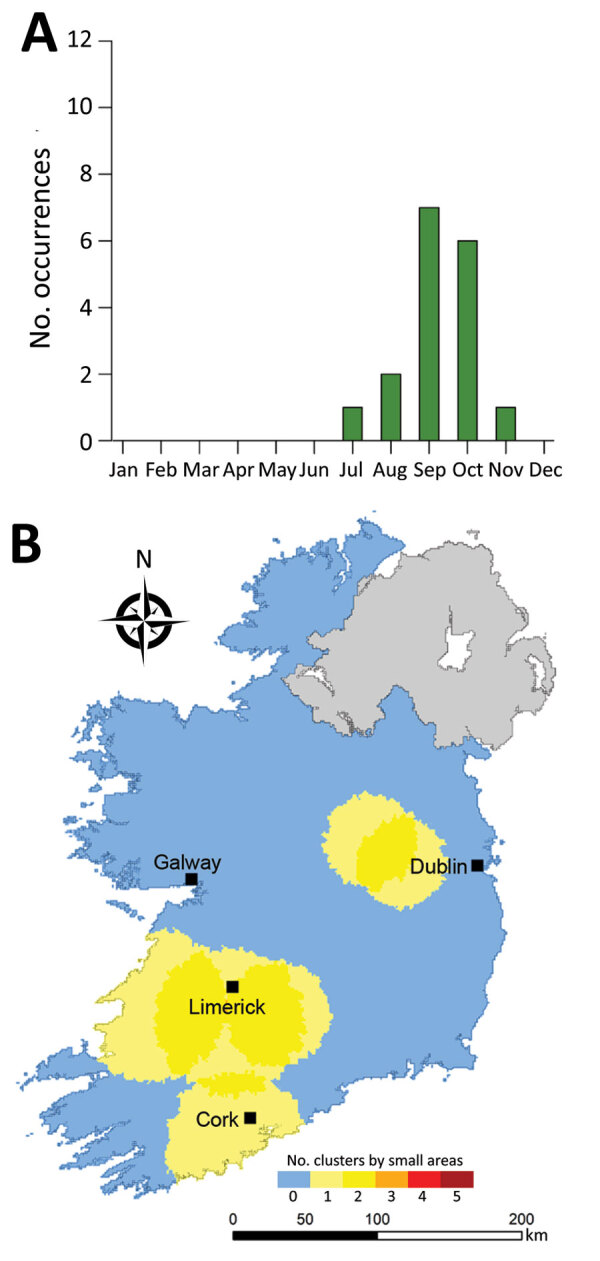
Monthly distribution of space-time clusters (A) and cluster recurrence index (0–5) within census small areas (B) for confirmed primary Shiga toxin–producing *Escherichia coli* (STEC) enteritis cases caused by STEC serogroup O157, Ireland, 2013–2017.

**Figure 10:**
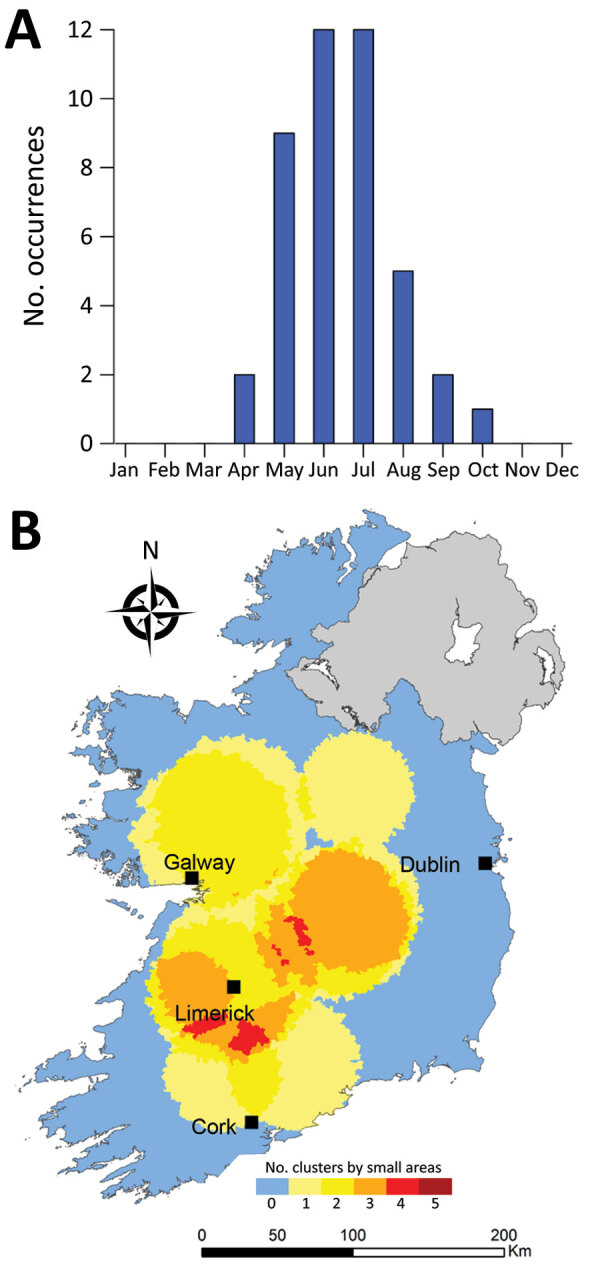
Monthly distribution of space-time clusters (A) and cluster recurrence index (0–5) within census small areas (B) for confirmed primary Shiga toxin–producing *Escherichia coli* (STEC) enteritis cases caused by STEC serogroup O26, Ireland, 2013–2017.

We observed much less space-time clustering (i.e., occurrence more geographically distributed) for STEC O157 infection than STEC O26 infection; Most STEC O157 clustering was low (1–2 clusters over the study period) in the south, south-west, and midlands zones (Figure 9). The spatial distribution and recurrence index of STEC O26 clusters mirrored those found for all confirmed STEC infections (Figure 10). The temporal window of serogroup-specific space-time clusters reflected the decomposed seasonal peak for both serogroups; STEC O157 clusters occurred more frequently in September–December, whereas STEC O26 clusters typically occurred in June–November.

**Figure 11:**
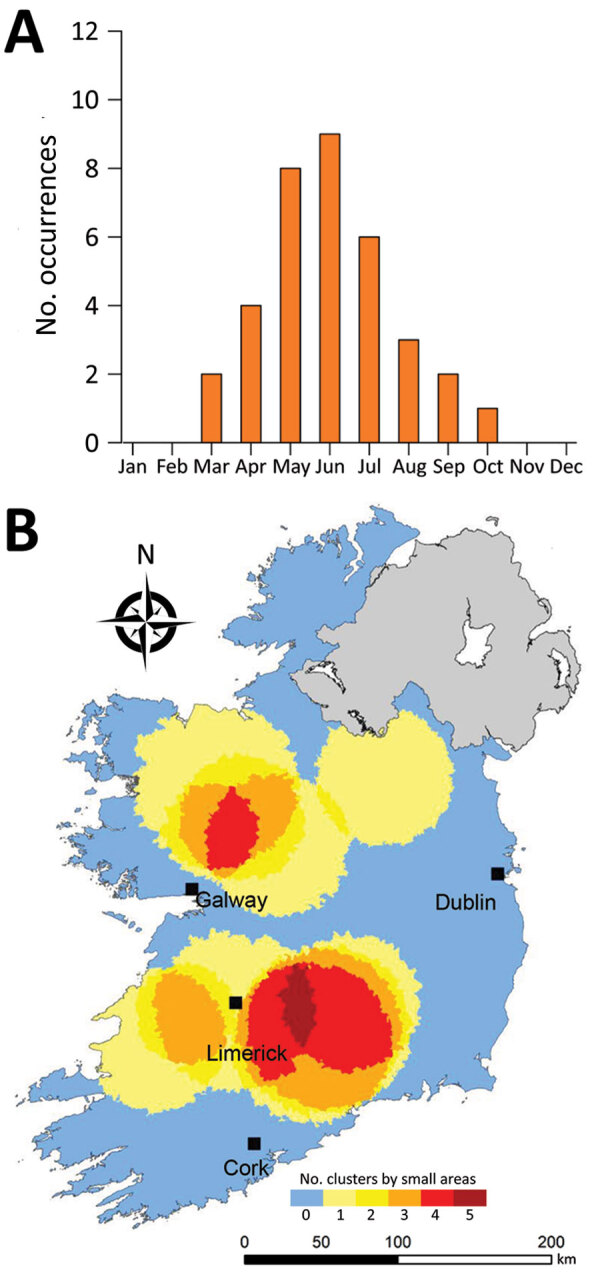
Monthly distribution of space-time clusters (A) and cluster recurrence index (0–5) within census small areas (B) for confirmed primary Shiga toxin–producing *Escherichia coli* (STEC) enteritis cases among patients <5 years of age, Ireland, 2013–2017.

**Figure 12:**
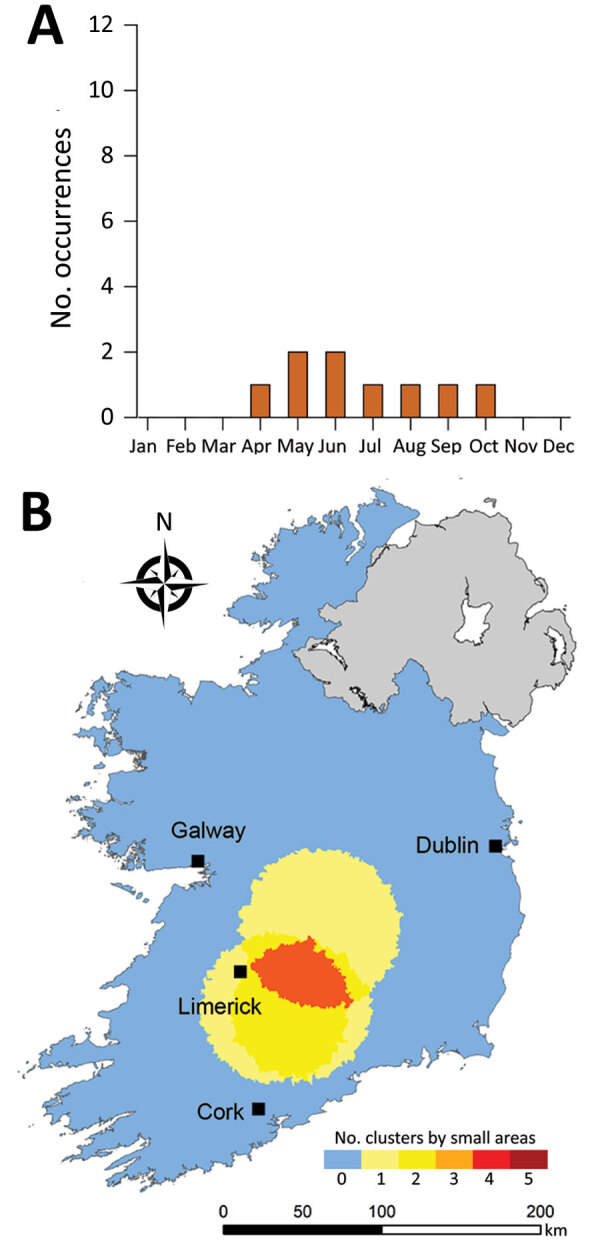
Monthly distribution of space-time clusters (A) and cluster recurrence index (0–5) within census small areas (B) for confirmed primary Shiga toxin–producing *Escherichia coli* (STEC) enteritis cases among patients >65 years of age, Ireland, 2013–2017.

We identified much of the western seaboard as a particularly high incidence region for the <5 year subpopulation (zones W, SW, S) (Figure 11), with a notable temporal clustering peak (April–May) and relatively broad temporal baseline (March–September). In contrast, we noted 3 space-time clusters within the >65 year subpopulation (Figure 12); all occurred in the south of the country (zone S), with no specific temporal period associated with these clusters.

## Discussion

The power of understanding spatial and temporal patterns of infection has long been recognized (*15*); identifying infection hot and cold spots and their time periods informs targeted surveillance and control interventions and is a precursor to increasingly complex epidemiologic analyses and risk factor attribution (*16*–*18*). Since approximately 2000, space-time scanning and geostatistical approaches have been increasingly recognized as powerful tools for endemic disease surveillance and early outbreak detection (*19*).

Overall, we identified 17 space-time clusters during the 5-year study period, ranging from 2 clusters during 2014 to 5 clusters during 2017. All analyses were of categorically sporadic infections; thus, the identification of distinct space-time clusters is noteworthy and underlines the potential utility of real-time or prospective space-time scanning as part of ongoing surveillance procedures. For example, Green et al. reported on the efficacy of using daily space-time statistics for 35 reportable communicable diseases in New York, New York, during 2014–2015 (*20*). The distribution of identified space-time clusters of sporadic STEC enteritis reveals high annual levels of persistence and variation in sporadic STEC infection in Ireland. We identified 3 distinct regions as exhibiting particularly high space-time cluster recurrence rates (Figure 6), namely southwest and east of Limerick city (zones SW, S, and SE), and northeast of Galway city (zone M), indicating the presence of persistent STEC reservoirs in these areas that cause regular exposure and transmission. 

Spatial autocorrelation of STEC clusters further highlights the disparity between rural and urban living. Sporadic cases were more frequently identified in rural areas where ≈37.3% of the populace reside (20.1% of rural SAs vs. 8.9% of urban) (*21*). We identified low-incidence clusters in major cities, including Cork and the greater Dublin area. These findings emphasize the association of rurality with STEC transmission; increased environmental exposure to pathogen sources coupled with enhanced transport of pathogens through untreated drinking water supplies, extreme weather events, and so on are likely to increase risk for exposure and subsequent infection (*22*). 

The relative proximity of large urban centers to the 3 identified high-recurrence regions may also point to narrow transitional zones between urban and populated-rural regions. Rural commuter belts that have inadequate municipal wastewater treatment or drinking supplies, in addition to relatively low levels of acquired immunity among children of young families residing within commuter belt regions, may contribute to this high spatial risk for transmission (*6*). National census statistics predict strongest population growth in peri-urban/commuter belt areas in Ireland (*21*), which are potentially at high risk for STEC infection incidence.

All 3 high-recurrence regions are predominantly underlain by karstified carboniferous limestone aquifers (*23*), which have previously been associated with the presence of STEC in private and small public drinking water supplies (*7*). The lack of space-time clustering found within the Greater Dublin area, which houses ≈39% of the national population (1.9 million persons) and is characterized by a spatially extensive urban commuter belt, consolidated bedrock, and a high level of water and wastewater infrastructure, seems to validate our hypotheses. Boudou et al. (2021) report that rates of space-time cluster recurrence of cryptosporidiosis from 2008 to 2017 followed similar patterns in the same 3 geographically distinct regions we identified; co-occurrence of STEC enteritis and cryptosporidiosis in Ireland requires further study (*24*).

Cumulative incidence rates of STEC infection exhibit a marked seasonal distribution; we identified peaks during late summer and early autumn, reflecting previously noted patterns of STEC shedding from zoonotic reservoirs and subsequent influx to the environment (*25*). Our findings, however, indicate a geographic and temporal disparity between the 2 primary serogroups, STEC O157 and STEC O26, with high-incidence geographic clusters of STEC O157 occurring more frequently in zones E and S. Previous work has identified associations between STEC O157 infection and persons residing in areas characterized by a higher density of cattle, private well usage, and domestic wastewater treatment systems (*6*), all of which are very common within spatial locations identified as HH clusters (*26*).

The <5 year age category has been associated with cases of STEC O26 infection, which has been characterized by an earlier annual infection peak in Ireland (*5*), implying age-specific peaks of infection. Garvey et al. (2016) reported a 2-month phase difference between STEC O26 (July) and STEC O157 (September) infections in Ireland; the difference was reported as significant in all (outbreak and sporadic) confirmed STEC infections (p<0.0001) and in sporadic cases only (p<0.0001) and possibly attributed to seasonal variation in infection exposure such as contact with primary animal reservoirs of infection (*5*). Significantly higher incidence rates were noted in children <5 years of age; previous studies attributed this pattern to an increased risk for direct contact with environmental sources of fecal matter (*27*) and lower standards of hygiene (*28*) within this subpopulation.

Cumulatively, young children (<5 years) and the older subpopulation (>65 years) accounted for 56.7% (n = 1,563) of confirmed sporadic infections. Both of these subpopulations are known to be immunologically vulnerable and exhibit higher incidence rates of infection and severe sequelae (*29*,*30*). Younger cohorts especially are at increased risk of infection caused by frequent contact with other children of a similar age, and also pose a risk as a source of infection associated with increased contact with adults, particularly among the 30–39 year age group (*31*,*32*). Prominent clustering of infection identified among the <5 and >65 year age groups, and the relative spatial heterogeneity of infection clusters, underscore the need for enhanced targeted surveillance measures, particularly in geographic areas characterized by a higher proportion of younger and older populations (*33*).

Appendix Additional information about sporadic Shiga toxin–producing *Escherichia coli* enteritis cases, Ireland, 2013–2017.
