# Supplementary material for: Spatiotemporal Dynamics of Sporadic Shiga Toxin–Producing Escherichia coli Enteritis, Ireland, 2013–2017
Source: Emerg Infect Dis. 2021 Sep;27(9):2421–33. doi: 10.3201/eid2709.204021 (PMC8386769; doi:10.3201/eid2709.204021)
Supplement: Appendix — Additional information about sporadic Shiga toxin–producing Escherichia coli enteritis cases, Ireland, 2013–2017. [file 20-4021-Techapp-s1.pdf]

Article DOI: https://doi.org/10.3201/eid2709.204021

Spatiotemporal Dynamics of Sporadic Shiga Toxin–Producing *Escherichia coli* Enteritis, Ireland, 2013–2017

Appendix

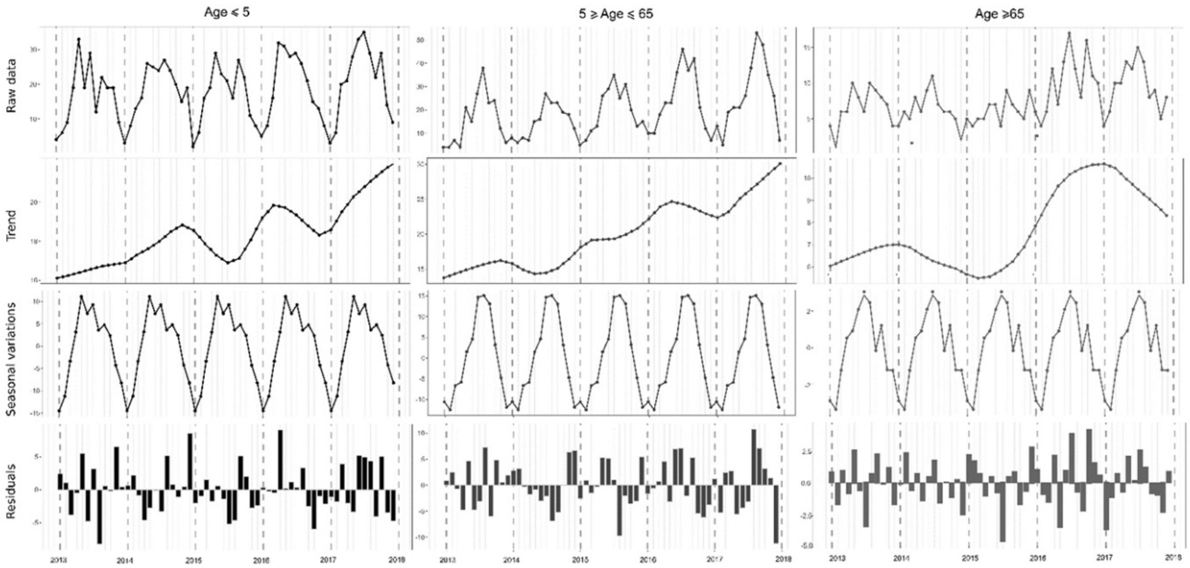

Appendix Figure 1. Seasonal decomposition of sporadic cases of Shiga toxin–producing *Escherichi coli* bacteria in Ireland, 2013–2017, delineated by relevant age subcategories.

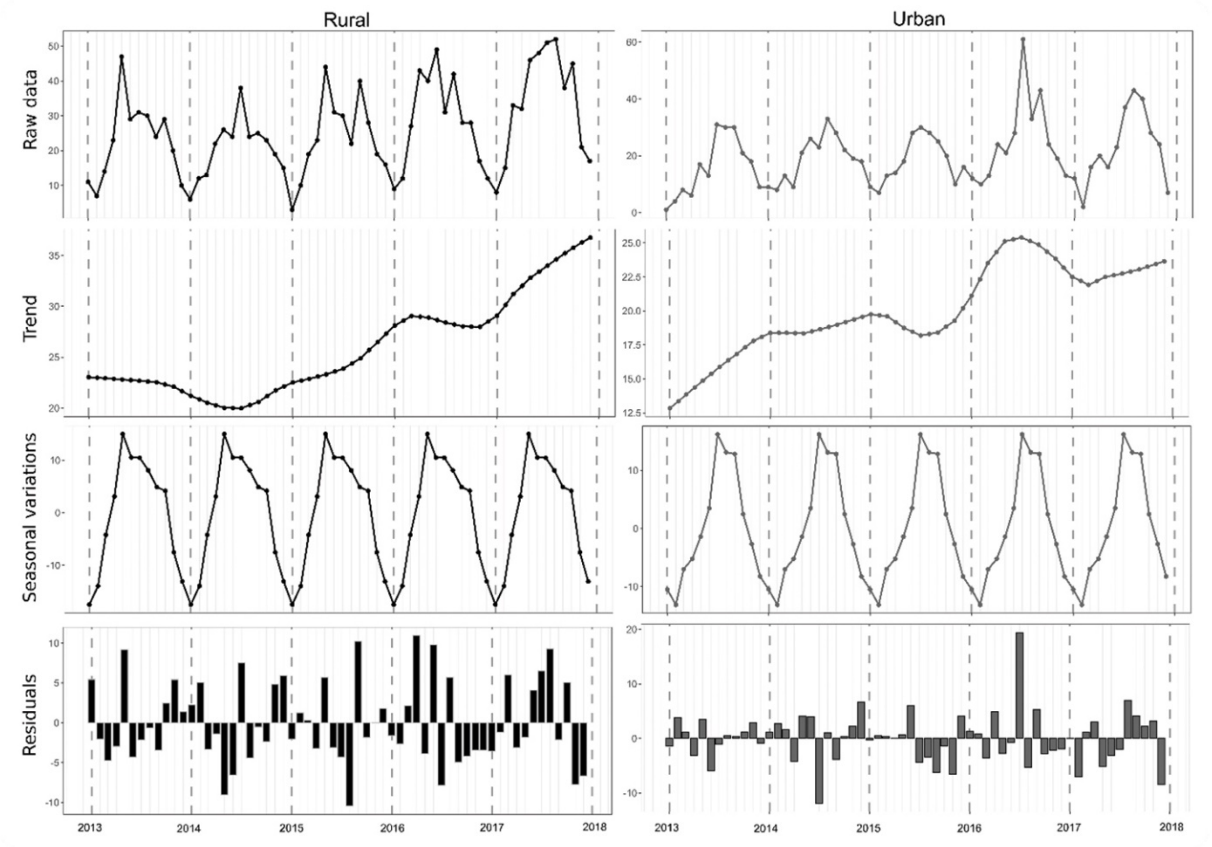

Appendix Figure 2. Seasonal decomposition of sporadic verocytotoxin-producing *Escherichia coli* cases in Ireland, 2013–2017, delineated by Central Statistics Office urban/rural classification.
